# Impact of quality control indicator system management program on neoadjuvant and adjuvant therapies for breast cancer: a comparative analysis

**DOI:** 10.3389/fonc.2026.1597546

**Published:** 2026-01-27

**Authors:** Silu Xu, Nianyang Ding, Lili Zhang, Sainan Hu, Meixin Ni, Haijuan Gu, Zeng Chen, Zhengzheng Xie, Nan Wu, Yuanyuan Wen, Panqi Fang, Xin Liu, Xinwei Dong, Yiming Jiang, Dan Yan

**Affiliations:** 1 Department of Pharmacy, Jiangsu Cancer Hospital & Jiangsu Institute of Cancer Research & The Affiliated Cancer Hospital of Nanjing Medical University, Nanjing, Jiangsu, China; 2 Department of Oncology, Jiangsu Cancer Hospital & Jiangsu Institute of Cancer Research & The Affiliated Cancer Hospital of Nanjing Medical University, Nanjing, Jiangsu, China; 3 Department of Pharmacy, Nantong Tumor Hospital/Affiliated Tumor Hospital of Nantong University, Nantong, Jiangsu, China; 4 Department of Information Office, Jiangsu Cancer Hospital & Jiangsu Institute of Cancer Research & The Affiliated Cancer Hospital of Nanjing Medical University, Nanjing, Jiangsu, China; 5 Beijing Shijitan Hospital Capital Medical University, Beijing, China

**Keywords:** anti-tumor drugs, breast cancer, neoadjuvant and adjuvant therapy, quality control indicators, standardized treatment

## Abstract

**Background:**

Breast cancer (BC) management in China faces significant challenges, particularly in terms of standardizing diagnosis and treatment protocols across various healthcare settings. The implementation of a quality control (QC) indicator system offers a potential solution to improve treatment consistency and outcomes. This study evaluated the impact of a QC indicator system on the standardization of neoadjuvant and adjuvant therapies for BC.

**Methods:**

A QC system was developed by an expert panel in alignment with China’s 2022 guidelines. A comparative study was conducted between Jiangsu Cancer Hospital (experimental, implementing PDCA-cycle management) and Nantong Tumor Hospital (control, routine practices). Key indicators included cTNM staging completeness, chemotherapy record standardization, and rational anti-tumor drug use. Additionally, clinical outcomes, patient-reported outcomes (PROs), and treatment adherence were assessed, alongside qualitative data derived from clinical records.

**Results:**

After the QC system was implemented, Jiangsu Cancer Hospital demonstrated significant improvements: cTNM staging completeness increased from 92.5% to 98.08%, chemotherapy record standardization improved by 66.65%, and rational drug use rose from 78% to 87.5% (all P<0.05). In contrast, no significant improvements were observed in the control hospital. The clinical outcomes and patient-reported symptoms also demonstrated favorable trends in the intervention hospital. Qualitative data revealed facilitators such as clearer treatment protocols and improved coordination, while challenges included increased documentation workload.

**Conclusions:**

The QC system effectively enhanced the standardization of neoadjuvant and adjuvant therapies for breast cancer, with improvements in clinical management and rational drug use. These results offer a scalable model for improving breast cancer management quality in other regions and institutions. Further research, including multi-center studies with extended follow-up, is recommended to validate these findings and explore the long-term impact of QC interventions on patient outcomes.

## 1 Introduction

Breast cancer (BC) is the most prevalent malignant tumor worldwide. In 2020, its global incidence was 2.26 million, accounting for approximately 11.7% of global cancer incidence (1). In 2022, BC ranked first among female malignant tumors in China, with an incidence of 357,200 and a mortality of 75,000 (2). Subsequently, this disease represents a major health threat to women in China. Despite ongoing improvements in the diagnosis and treatment of BC, disparities in the quality of care and inconsistencies in diagnostic and treatment protocols across different regions persist. Enhancing the overall survival rate of BC patients in China is intrinsically linked to standardizing diagnostic and treatment protocols across medical institutions. Achieving such standardization requires the implementation of robust medical quality control (QC) measures and effective management systems.

Given the growing burden of malignant tumors in China, the Healthy China 2030 Plan has explicitly outlined the objectives to achieve comprehensive health management for chronic diseases across the entire population and at all stages of life by 2030. A key goal of this plan is to achieve a 15% increase in the overall 5-year survival rate for cancer (3). The 2019 Opinions on Implementing the Healthy China Action report by the State Council further elaborated on the objectives of the Healthy China 2030 Plan by setting specific targets for the 5-year cancer survival rate, initially targeting a minimum increase of 43.3% by 2022 and 46.6% by 2030 (4). Despite these efforts, significant challenges persist, such as disparities in diagnostic and treatment quality, suboptimal implementation of clinical guidelines, and limited sharing of diagnostic and treatment information. Addressing these issues is critical to improving the cancer survival rate in China. Accordingly, the Healthy China 2030 Plan emphasizes the importance of improving the quality and standardization of cancer diagnosis and treatment. To achieve this, it advocates for the establishment of a healthcare quality management and control system that aligns with international standards while integrating distinct Chinese characteristics (3).

In 2012, with the approval of the National Health Commission (NHC), the National Cancer Center of China (NCC) established the National Cancer Quality Control Center (NCQCC) tasked with enhancing the quality, standardization, and homogeneity of cancer diagnosis and treatment in China. On August 3, 2018, the NCC and the NCQCC established the first single-disease quality control expert committee: the NCQCC-Breast Cancer Expert Committee. In September 2019, the NCC launched the Pilot Project on Quality Control of Standardized Diagnosis and Treatment of Breast Cancer. In December 2020, the NCC identified 200 hospitals to serve as pilot centers, including the Cancer Hospital of the Chinese Academy of Medical Sciences, Peking Union Medical College, and Jiangsu Cancer Hospital. In March 2022, the NCQCC-Breast Cancer Expert Committee released the Quality Control Index for Standardized Diagnosis and Treatment of Breast Cancer in China (2022 Edition) (QCISDTBC-2022) (5). This index comprises 20 quality control and four quality management indicators, including metrics such as the evaluation rate of clinical tumor-node-metastasis (TNM) staging before the initial treatment of BC patients. Additionally, the index encompasses various aspects of care such as diagnosis, pathology, surgery, drug therapy, and radiotherapy, thereby providing a foundation for advancing QC efforts. However, practical research remains limited regarding the alignment of this indicator system with actual clinical practice and how these indicators can be utilized to improve the diagnosis and treatment of BC.

This study aims to enhance the QC system for BC diagnosis and treatment and to establish an effective management model by leveraging Jiangsu Cancer hospital’s role as one of the initial pilot centers for the standardized quality control of BC diagnosis and treatment. The study aims to implement targeted QC measures in neoadjuvant and adjuvant therapies for BC, with emphasis on pre-treatment staging, along with pathological, surgical, and drug therapy quality controls. The goal is to develop clinically applicable quality control indicators and incorporate them into a dedicated management program to improve the standardization and homogenization of BC care.

## 2 Methods

### 2.1 Establishment of quality control indicator system

**Figure 1:**
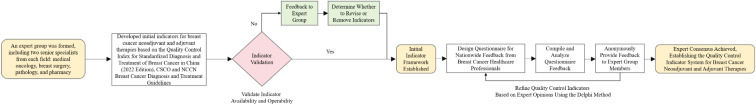
Illustration of the process of developing the quality control indicator system for breast cancer neoadjuvant and adjuvant therapies.

To develop a QC indicator system that aligns with clinical practice for standardized BC diagnosis and treatment in neoadjuvant and adjuvant settings, an expert panel was assembled. The panel comprised two senior specialists from each field of the following fields: medical oncology, breast surgery, pathology, and pharmacy. The expert group first developed a conceptual framework through expert consultations, based on QCISDTBC-2022, the Chinese Society of Clinical Oncology (CSCO), and the National Comprehensive Cancer Network (NCCN) Breast Cancer Diagnosis and Treatment Guidelines (5–7). Subsequently, a comprehensive questionnaire was developed and distributed to collect extensive feedback from medical professionals nationwide. This iterative process culminated in the development of the final QC indicator system for BC neoadjuvant and adjuvant therapies. The detailed methodology for developing the system is illustrated in **Figure 1**.

### 2.2 Quality control indicator system management program

#### 2.2.1 Baseline data collection and identification of key intervention indicators

Baseline data were retrospectively extracted from the hospital information system (HIS) for breast cancer patients who underwent neoadjuvant or adjuvant therapy at Jiangsu Cancer Hospital between July and September 2022. Extracted variables included patient identifiers, demographic characteristics, tumor staging, pathological findings, treatment regimens, dosing details, and documentation related to predefined QC indicators. To ensure data integrity and completeness, several quality assurance procedures were implemented. First, two trained researchers independently extracted and evaluated all QC indicators using a back-to-back review approach. Second, discrepancies between reviewers were systematically compared and resolved through consensus discussion, with arbitration by the multidisciplinary expert panel when necessary. Third, source data validation was performed by cross-checking indicator values against original electronic medical records, chemotherapy order systems, pathology reports, and pharmacy dispensing records. Finally, completeness checks were conducted, and records with missing critical variables required for indicator calculation were flagged and re-reviewed. Incomplete cases were excluded from indicator-specific analyses where appropriate. Subsequently, indicators associated with significant deficiencies in our hospital’s neoadjuvant and adjuvant BC therapies were identified and prioritized as key intervention targets. To improve the systematic quality, a Plan-Do-Check-Act (PDCA) cycle was implemented to address these targets.

#### 2.2.2 Program implementation

The management of the key intervention indicators using the PDCA cycle involved the following protocols: 1) Plan: Based on the baseline data analysis, a multidisciplinary team (MDT) was established at our hospital, comprising the Medical Affairs Department, Quality Control Office, Nursing Department, Pharmacy Department, Information Department, and relevant clinical departments. This team formed the BC Neoadjuvant and Adjuvant Therapy Management Group. The group identified the causes of suboptimal QC indicators and developed strategies to address these issues using discussion sessions and fishbone diagrams. 2) Do: The deficiencies in the identified QC indicators associated with the neoadjuvant and adjuvant BC therapies were addressed using specific measures, which were iteratively improved since December 2022. 3) Check: The QC indicator data for BC patients who underwent either neoadjuvant or adjuvant therapy at our hospital from September to November 2023 were analyzed to assess the effectiveness of the implemented measures. This analysis aimed to determine whether significant improvements had been achieved and to identify any remaining issues in the execution of the quality improvement initiatives. 4) Act (A): Effective measures were standardized and incorporated into institutional work protocols to ensure consistency in future applications. These standardized measures were also prepared for broader dissemination to support the widespread adoption of improved practices.

#### 2.2.3 Post-intervention data collection and data quality assurance

To ensure comparability between baseline and post-intervention assessments, identical data extraction, verification, and validation procedures were applied to post-intervention data. Two independent researchers again conducted parallel data abstraction, followed by cross-verification and expert adjudication of discrepancies. Indicator definitions, denominator specifications, and inclusion/exclusion criteria were kept strictly consistent across time points.

#### 2.2.4 Comparative analysis

**Figure 2:**
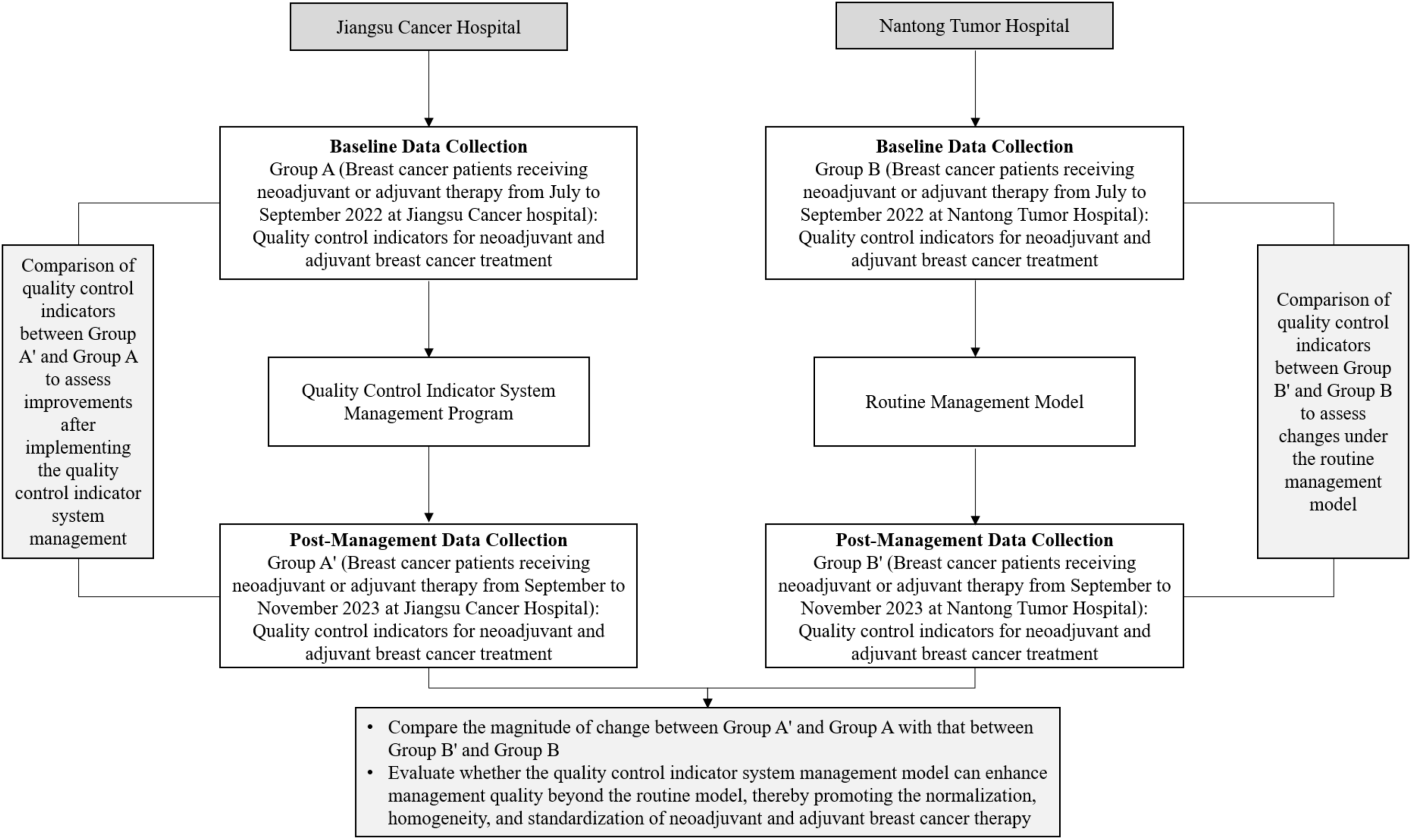
Comparative study workflow of management models at jiangsu cancer hospital and nantong tumor hospital.

A comparative study was conducted between Jiangsu Cancer Hospital and Nantong Tumor Hospital, as illustrated in **Figure 2**. Jiangsu Cancer Hospital was designated as the experimental unit, where a BC quality control indicator system (QCIS) management program was implemented. Nantong Tumor Hospital served as the control unit, adhering to routine management practices. Before the implementation of the quality management program, baseline data on relevant quality control indicators were collected from BC patients undergoing either neoadjuvant or adjuvant therapy at both hospitals between July and September 2022. The management interventions were initiated in December 2022 and continued throughout the study period, with the post-management data collected from September to November 2023 to evaluate improvements. Data extraction and indicator calculations followed the same standardized procedures in both institutions to enhance internal validity. To further compare the effect of the QCIS program between hospitals, changes in indicator rates from baseline to post-management (Δ rate) were calculated for each hospital. Differences in Δ rates between Jiangsu Cancer Hospital and Nantong Tumor Hospital (ΔΔ) were compared using z-tests based on binomial variance, analogous to a difference-in-differences approach. This study was approved by the Ethics Committee of Jiangsu Cancer Hospital and Nantong Tumor Hospital, China.

### 2.3 Clinical outcomes, patient-reported outcomes, and treatment adherence

In addition to predefined QC indicators, exploratory clinical outcomes and patient-reported outcomes (PROs) were retrospectively collected to provide insights into the management program’s clinical impact. Clinical outcomes included the pathological complete response (pCR) rate and the 2-year disease-free survival (DFS) rate. pCR was defined as the absence of residual invasive cancer in the breast and axillary lymph nodes at surgery following neoadjuvant therapy. The 2-year DFS rate was the proportion of patients free from recurrence or death within 24 months of definitive surgery.

Patient-reported symptoms were extracted from routine follow-up documentation. Symptoms included fatigue, nausea/vomiting, diarrhea, precordial discomfort, and peripheral neuropathy, recorded based on patient self-report during treatment or follow-up. Other treatment-related toxicities were assessed using clinical documentation and lab test results. Myelosuppression was defined as the occurrence of grade ≥2 cytopenia, including neutropenia, anemia, or thrombocytopenia, according to the Common Terminology Criteria for Adverse Events (CTCAE) version 5.0. Liver damage was defined as grade ≥2 elevations in liver function parameters (e.g., ALT, AST, or bilirubin) based on CTCAE v5.0 (8).

Treatment adherence was evaluated as the proportion of patients who completed all planned cycles of neoadjuvant or adjuvant chemotherapy or targeted therapy without premature discontinuation for non-medical reasons, such as patient refusal or loss to follow-up. Endocrine therapy was not included in the adherence assessment due to its prolonged duration. These exploratory indicators were analyzed descriptively and not included in the primary QCIS but provided additional patient-centered and clinical context for interpreting the management program’s potential effects.

### 2.4 Qualitative data collection

In conjunction with the quantitative assessment, qualitative information was collected to provide context for clinicians’ and patients’ experiences during the implementation period. Narrative comments documented in routine clinical records—such as follow-up notes, MDT communication records, and internal management logs—were reviewed retrospectively to identify recurring themes related to perceived barriers and facilitators. For clinicians, relevant narrative entries were extracted from meeting summaries, MDT notes, internal communications, and documentation linked to quality review activities. For patients, narrative feedback was sourced from follow-up notes and treatment-related encounters in the hospital information system. All text excerpts were anonymized before review. A thematic synthesis approach was employed to summarize recurring patterns. These qualitative observations were designed to complement the quantitative indicators by offering additional insights into real-world experiences and contextual factors that influenced the implementation of the management program.

## 3 Results

### 3.1 Quality control indicator system for neoadjuvant and adjuvant BC therapies

The expert panel identified 20 QC indicators associated with peri-operative treatment of BC, encompassing diagnostic aspects, pathological factors, surgical procedures, and pharmacotherapy. The availability and applicability of the initial indicators were validated by data analysts from Jiangsu Cancer Hospital and Nantong Tumor Hospital using information databases from their respective institutions. This process identified 18 accessible indicators. Subsequently, a survey questionnaire was designed based on these 18 indicators and distributed to healthcare professionals nationwide involved in the standardized diagnosis and treatment of BC. A total of 793 individuals completed the survey, comprising 424 physicians, 359 pharmacists, 6 nurses, 2 public health specialists, and 2 experts in statistics and computer science. Analysis of the survey data revealed that 97.98% of the respondents affirmed that utilization of quality control indicators has the potential to enhance the standardization of BC diagnosis and treatment. Additionally, focus areas among respondents included pharmacotherapy indicators (94.96%), diagnostic indicators (87.91%), pathological indicators (83.63%), surgical indicators (76.7%), radiotherapy indicators (68.26%), imaging indicators (64.74%), and indicators associated with average cost per treatment session (55.16%).

The ranking of the diagnostic, surgical, and pathological quality control indicators based on expert-perceived importance, from highest to lowest was as follows: 1) Completion rate of clinical TNM (cTNM) staging before treatment; 2) Pathological diagnosis rate before anti-tumor drug therapy; 3) Sentinel lymph node biopsy rate in patients with early-stage BC (clinical stage T_1-2_N_0_M_0_); 4) Post-operative human epidermal growth factor receptor 2 (HER2) (2+) fluorescence *in situ* hybridization (FISH) testing rate; 5) Proportion of neoadjuvant therapy in surgical patients with clinical stage III BC (excluding T_3_N_1_M_0_); 6) Pathological evaluation report rate after neoadjuvant therapy for BC. For the pharmacotherapy quality control indicators, the expert-perceived ranking based on importance from highest to lowest was as follows: 1) Standardization rate of chemotherapy records; 2) Proportion of patients with hormone receptor (estrogen receptor (ER), progesterone receptor (PR))-positive status among those receiving adjuvant endocrine therapy post-BC surgery; 3) Proportion of HER2-positive patients among those receiving anti-HER2 targeted therapy in neoadjuvant and adjuvant treatment for BC; 4) Rate of rational use of anti-tumor drugs; 5) Expenditure on anti-tumor drugs; 6) Frequency and reporting rate of adverse reactions to anti-tumor drugs.

Following the collection of feedback from the questionnaire survey, the panel used the Delphi method to consult with the experts to refine the quality control indicators. This process resulted in the development of a comprehensive quality control indicator system for neoadjuvant and adjuvant therapies for BC. The final system is outlined in detail below. The calculation methods for the 10 quality control indicators are shown in **Equations 1**–**10**.

I. Proportion of complete cTNM staging of BC patients before the initial treatment.

1. Definition: the proportion of BC patients who are diagnosed with complete cTNM staging before the initial treatment among all the BC patients receiving initial treatment.

2. Calculation formula: Formula [1].

(1) $$\begin{array}{l} \text{Proportion of BC patients undergoing neoadjuvant or adjuvant therapies with complete cTNM staging before the initial treatment}=\\ \frac{\text{Number of BC patients undergoing neoadjuvant or adjuvant therapies with complete cTNM staging before the initial treatment}}{\text{Total number of BC patients undergoing neoadjuvant or adjuvant therapies who received initial treatment in the same time period}}\;\times \;100\% \end{array}$$

3. Patient population: Hospitalized patients.

4. Rationale: This index reflects a thorough assessment of the disease before treatment, serving as the basis of standardized cancer treatment.

5. Improvement indices: Increase in proportion.

6. References for this index: QCISDTBC-2022 and American Joint Committee on Cancer (AJCC) Cancer Staging Manual (8th edition) (5, 9).

II. Proportion of pathological diagnosis of BC patients before the initial anti-tumor treatment.

1. Definition: the proportion of BC patients who received a pathological diagnosis before the initial anti-tumor treatment among all the BC patients receiving initial anti-tumor treatment.

2. Calculation formula: see Formula [2].

(2) $$\begin{array}{l} \text{Proportion of BC patients with a pathological diagnosis before the intitial anti}-\text{tumor reatment during neoadjuvant or adjuvant treatment}=\\ \frac{\text{Number of BC patients with pathological diagnosis before the initial anti}-\text{tumor treatment during neoadjuvant or adjuvant treatment}}{\text{Total number of BC patients undergoing neoadjuvant or adjuvant therapies who received initial anti}-\text{tumor treatment in the same time period}}\;\times \;100\% \end{array}$$

3. Patient population: Hospitalized patients.

4. Rationale: A definitive pathological diagnosis is the foundation for selecting a comprehensive cancer treatment plan.

5. Improvement indices: Increase in proportion.

6. References for this index: QCISDTBC-2022 (5).

III. Proportion of BC patients with clinical stage III BC receiving neoadjuvant therapy before surgery.

1. Definition: Proportion of BC surgery patients with clinical stage III BC (excluding T_3_N_1_M_0_) who received neoadjuvant therapy before surgery, compared to the total number of clinical stage III BC (excluding T_3_N_1_M_0_) surgery patients admitted during the same period.

2. Calculation formula: see Formula [3].

(3) $$\begin{array}{l} \text{Proportion of BC patients with clinical stage III BC who received neoadjuvant therapy before surgery}=\\ \frac{\text{Number of clinical stage III BC }\left(\text{excluding T}3\text{N}1\text{M}0\right)\text{ patients who received neoadjuvant therapy before surgery}}{\text{Total number of clinical stage III BC }\left(\text{excluding T}3\text{N}1\text{M}0\right)\text{ surgery patients in the same time period}}\;\times \;100\% \end{array}$$

3. Patient population: Hospitalized patients.

4. Rationale: This index reflects the degree of standardization in the treatment of patients with locally advanced BC.

5. Improvement indices: Increase in proportion.

6. References for this index: QCISDTBC-2022 (5).

IV. Surgical rate of sentinel lymph node biopsy in patients with early-stage BC (clinical stage T_1-2_N_0_M_0_).

1. Definition: Proportion of early-stage BC patients (clinical stage T_1-2_N_0_M_0_) who underwent sentinel lymph node biopsy, compared to the total number of early-stage BC surgery patients during the same period.

2. Calculation formula: see Formula [4].

(4) $$\begin{array}{l} \text{Proportion of sentinel lymph node biopsy in patients with early}-\text{stage BC}=\\ \frac{\text{Number of early}-\text{stage BC }\left(\text{clinical stage T}1-2\text{N}0\text{M}0\right)\text{ patients undergoing sentinel lymph node biopsy}}{\text{Total number of early}-\text{stage BC surgery patients in the same time period}}\;\times \;100\% \end{array}$$

3. Patient population: Hospitalized patients.

4. Rationale: In early-stage BC, axillary lymph node metastasis occurs in only about 20% of patients. For the majority who do not have axillary metastasis, traditional axillary lymph node dissection offers no survival benefit and may cause serious complications, such as upper limb lymphedema, which can significantly impact postoperative quality of life. Therefore, sentinel lymph node biopsy is recommended for patients with clinically early-stage, axillary lymph node-negative breast cancer (T_1-2_N_0_M_0_).

5. Improvement indices: Increase in proportion.

6. References for this index: QCISDTBC-2022 (5).

V. Reporting rate of chemotherapy response after neoadjuvant chemotherapy in BC patients.

1. Definition: Number of breast cancer patients with pathological reports documenting chemotherapy response after neoadjuvant chemotherapy, compared to the total number of patients who underwent surgery following neoadjuvant chemotherapy during the same period.

2. Calculation formula: see Formula [5].

(5) $$\begin{array}{l} \text{Reporting rate of chemotherapy response after neoadjuvant chemotherapy in BC patients}=\\ \frac{\text{Number of BC patients with pathological reports documenting chemotherapy response after neoadjuvant chemotherapy}}{\text{Total number of BC patients undergoing surgery after neoadjuvant chemotherapy in the same time period}}\times \;100\% \end{array}$$

3. Patient population: Hospitalized patients.

4. Rationale: Pathological response after chemotherapy is essential for assessing treatment effectiveness and guiding further clinical decisions.

5. Improvement indices: Increase in proportion.

6. References for this index: None.

VI. Proportion of hormone receptor-positive patients among BC patients who received adjuvant endocrine therapy after surgery.

1. Definition: Proportion of hormone receptor-positive BC patients receiving endocrine therapy after surgery, relative to the total number of breast cancer patients undergoing adjuvant endocrine therapy during the same period.

2. Calculation formula: see Formula [6].

(6) $$\begin{array}{l} \text{Proportion of hormone receptor}-\text{positive patients among BC patients who received adjuvant endocrine therapy after surgery}=\\ \frac{\text{Number of hormone receptor}-\text{positive BC patients receiving endocrine therapy after surgery}}{\text{Total Number of breast cancer patients receiving adjuvant endocrine therapy after surgery in the same time period}}\;\times \;100\% \end{array}$$

3. Patient population: Hospitalized patients.

4. Rationale: This index reflects the standardization of postoperative endocrine therapy in BC.

5. Improvement indices: Increase in proportion.

6. References for this index: None.

VII. Proportion of HER2 positive patients among BC patients who received anti-HER2 targeted therapy during neoadjuvant or adjuvant treatment.

1. Definition: Proportion of HER2 positive BC patients receiving HER2 targeted therapy during neoadjuvant or adjuvant treatment, relative to the total number of BC patients undergoing neoadjuvant or adjuvant treatment with HER2 targeted therapy during the same period.

2. Calculation formula: see Formula [7].

(7) $$\begin{array}{l} \text{Proportion of HER}-2\text{ positive patients among breast cancer patients who received anti}-\text{HER}-2\text{ targeted therapy during neoadjuvant or adjuvant treatment}=\\ \frac{\text{Number of HER}-2\text{ positive patients receiving HER}-2\text{ targeted therapy during neoadjuvant or adjuvant treatment for breast cancer}}{\text{Total Number of cases receiving neoadjuvant and adjuvant HER}2-\text{targeted therapy in breast cancer}}\;\times \;100\% \end{array}$$

3. Patient population: Hospitalized patients.

4. Rationale: This index determines whether all perioperative breast cancer patients receiving HER2-targeted therapy meet the indications, reflecting the standardization of HER2-targeted drug use.

5. Improvement indices: Increase in proportion.

6. References for this index: None.

VIII. Standardization rate of chemotherapy records in BC patients.

1. Definition: The proportion of BC patients receiving neoadjuvant or adjuvant chemotherapy who have recorded their chemotherapy regimen, drug dosage, and treatment duration, relative to the total number of patients receiving neoadjuvant or adjuvant chemotherapy during the same period.

2. Calculation formula: see Formula [8].

(8) $$\begin{array}{l} \text{Standardization rate of neoadjuvant and adjuvant chemotherapy records in BC patients}=\\ \frac{\text{Number of BC cases with recorded neoadjuvant and adjuvant chemotherapy regimens},\text{ chemotherapy doses},\text{ and chemotherapy duration}}{\text{otal Number of BC patients receiving neoadjuvant or adjuvant chemotherapy in the same time period}}\;\times \;100\% \end{array}$$

3. Patient population: Hospitalized patients.

4. Rationale: Chemotherapy regimens and drug dosages are key indicators of chemotherapy, serving as important reference points for assessing the standardization of patient treatment and formulating subsequent treatment plans.

5. Improvement indices: Increase in proportion.

6. References for this index: QCISDTBC-2022 (5).

IX. Rate of rational use of anti-tumor drugs in BC patients.

1. Definition: The proportion of BC patients undergoing neoadjuvant or adjuvant therapy with rational use of anti-tumor drugs, relative to the total number of such cases evaluated during the same period. Rational use of antitumor drugs includes the appropriate selection of regimens, correct chemotherapy dosages, accurate molecular targeted drug dosages, proper use of drug solvents, appropriate sequencing of chemotherapy agents, and the correct duration of neoadjuvant or adjuvant therapy.

2. Calculation formula: see Formula [9].

(9) $$\begin{array}{l} \text{Rate of rational use of anti}-\text{tumor drugs in BC patients undergoing neoadjuvant or adjuvant therapy}=\\ \frac{\text{Number of BC patients undergoing neoadjuvant or adjuvant therapy with the rational use of anti}-\text{tumor drugs}}{\text{Total number of BC patients undergoing neoadjuvant and adjuvant therapy with anti}-\text{tumor drug use reviewed in the same time period}}\;\times \;100\% \end{array}$$

3. Patient population: Hospitalized patients.

4. Rationale: This index reflects the standardization of drug therapy for BC.

5. Improvement indices: Increase in proportion.

6. References for this index: None.

X. Incidence of adverse reactions to anti-tumor drugs in BC patients.

1. Definition: The proportion of BC patients undergoing neoadjuvant or adjuvant therapy who experienced adverse reactions to antitumor drugs, relative to the total number of BC patients undergoing neoadjuvant or adjuvant therapy with antitumor drugs during the same period.

2. Calculation formula: see Formula [10].

(10) $$\begin{array}{l} \text{Incidence of adverse reactions in BC patients undergoing neoadjuvant or adjuvant therapy}=\\ \frac{\text{Number of BC patients undergoing neoadjuvant or adjuvant therapy who experienced adverse reactions to anti}-\text{tumor drugs}}{\text{Total number of BC patients undergoing neoadjuvant or adjuvant therapy with anti}-\text{tumor drugs during the same period}}\;\times \;100\% \end{array}$$

3. Patient population: Hospitalized patients.

4. Rationale: This index reflects the frequency of adverse reactions to anti-tumor drugs in BC patients undergoing neoadjuvant or adjuvant therapy, providing insight into the safety and tolerability of the treatment regimen.

5. References for this index: None.

### 3.2 Baseline data analysis

Analysis of the baseline data comprised 400 BC patients who underwent either a neoadjuvant or adjuvant therapy: this number involved 200 BC patients from Jiangsu Cancer Hospital (81 patients received neoadjuvant therapy and 119 patients received adjuvant therapy) and another 200 BC patients from Nantong Tumor Hospital (31 patients received neoadjuvant therapy and 169 patients received adjuvant therapy). The baseline quality control indicator data for BC from the two hospitals are presented in **Table 1**.

**Table 1:** Comparison of breast cancer quality control indicators at jiangsu cancer hospital and nantong tumor hospital.

Quality control indicator	Jiangsu cancer hospital		Nantong tumor hospital		Δ (Jiangsu cancer hospital)	Δ (Nantong tumor hospital)	P forΔΔ
	Baseline data (group A: n=200)	Post-management data (group A’: n=208)	Baseline data (group B: n=200)	Post-management data (group B’: n=200)			
BC patients receiving neoadjuvant therapy	81	39	31	45	–	–	–
BC patients receiving adjuvant therapy	119	169	169	155	–	–	–
Proportion of complete cTNM staging of BC patients before the initial treatment	92.50% (185/200)	98.08% (204/208)†	98.00% (196/200)	98.00% (196/200)	+5.58%	0.00%	0.027
Proportion of pathological diagnosis of BC patients before the initial anti-tumor treatment	100.00% (200/200)	100.00% (208/208)	99.50% (199/200)	99.50% (199/200)	0.00%	0.00%	1.000
Proportion of BC patients with clinical stage III receiving neoadjuvant therapy before surgery ^a^	71.43% (20/28)	57.89% (11/19)	29.73% (11/37)	39.39% (13/33)	-13.53%	+9.66%	0.202
Surgical rate of sentinel lymph node biopsy in patients with early-stage BC (clinical stage T_1-2_N_0_M_0_) ^b^	90.80% (79/87)	74.79% (89/119)†	74.79% (89/119)	44.44% (44/99)†	-16.01%	-30.55%	0.078
Reporting rate of chemotherapy response after neoadjuvant chemotherapy in BC patients ^c^	71.60% (58/81)	56.41% (22/39)	51.61% (16/31)	40.00% (18/45)	-15.19%	-11.61%	0.810
Proportion of hormone receptor-positive patients among BC patients who received adjuvant endocrine therapy after surgery ^d^	100.00% (13/13)	100.00% (7/7)	100.00% (15/15)	100.00% (18/18)	0.00%	0.00%	1.000
Proportion of HER2 positive patients among BC patients who received anti-HER2 targeted therapy during neoadjuvant or adjuvant treatment ^e^	100.00% (62/62)	100.00% (41/41)	100.00% (51/51)	100.00% (73/73)	0.00%	0.00%	1.000
Standardization rate of chemotherapy records in BC patients	4.50% (9/200)	71.15% (148/208)†	92.00% (184/200)	76.00% (152/200)†	+66.65%	-16.00%	<0.001
Rate of rational use of anti-tumor drugs in BC patients	78.00% (156/200)	87.50% (182/208)†	88.50% (177/200)	79.00% (158/200)	+9.50%	-9.50%	<0.001
Incidence of adverse reactions to anti-tumor drugs in BC patients	68.50% (137/200)	62.98% (131/208)	58.00% (116/200)	63.00% (126/200)	-5.52%	+5.00%	0.120

BC, breast cancer; TNM, tumor-node-metastasis; HER2, human epidermal growth factor receptor 2.

^a^The denominator was patients with clinical stage III breast cancer (excluding T_3_N_1_M_0_) who underwent surgery during the same period.

^b^The denominator was patients with early-stage breast cancer (clinical stage T_1–2_N_0_M_0_) who underwent surgery during the same period.

^c^The denominator was patients who underwent surgery following neoadjuvant chemotherapy during the same period.

^d^The denominator was patients who received adjuvant endocrine therapy during the same period.

^e^The denominator was patients who received neoadjuvant or adjuvant HER2-targeted therapy during the same period.

Unless otherwise specified, proportions were calculated using the total number of included patients in each group as the denominator.

† Significant (P < 0.05) compared to baseline data.

Δ represents the absolute change in proportion between post-management and baseline within each hospital.

P for ΔΔ represents the two-sided P value for the difference-in-differences between Jiangsu Cancer Hospital and Nantong Tumor Hospital, calculated using a Z test based on the standard error of the difference between two independent proportion changes.

Analysis of the baseline data revealed several issues with the neoadjuvant and adjuvant treatment approach for BC: 1. The proportion of complete cTNM staging of BC patients before initial treatment requires significant improvement, given challenges such as lack of TNM staging in the hospitalization data of some patients and diagnosis of T_x_N_x_M_x_. 2. The proportion of BC patients with clinical stage III receiving neoadjuvant therapy before surgery was relatively low since most of these patients had surgery at other hospitals and post-operatively sought further treatment at our hospital. 3. The standardization rate of chemotherapy records in BC patients was low. Although the majority of chemotherapy records included the regimen and dosage, there were cases with missing chemotherapy dosage details, or inadequate details on the specific neoadjuvant/adjuvant treatment plan used. In many cases, the administration intervals of the treatment regimen and the specific timing of anti-tumor drug administration were inadequately documented. 4. The rational use rate of anti-tumor drugs in BC patients was suboptimal. Notably, there were occurrences of off-label use of pegylated liposomal doxorubicin (PLD) in neoadjuvant and adjuvant therapies. Additionally, there were cases where the selection of antitumor drug regimens was deemed inappropriate or where the drug dosages did not align with the clinical guidelines, thereby raising concern about the rationality of the pharmacological approach.

Based on the baseline data analysis from our hospital and the feasibility analysis of indicator interventions, the expert panel concluded that the following indicators should be included in the BC quality control indicator system management program: Proportion of complete cTNM staging of BC patients before the initial treatment, standardization rate of chemotherapy records in BC patients, and rate of rational use of anti-tumor drugs in BC patients.

### 3.3 Program implementation

#### 3.3.1 Causal analysis

The expert panel used discussion sessions and fishbone diagrams to evaluate the causes of the deficiencies in the key intervention indicators at Jiangsu Cancer Hospital. Their analysis revealed the following factors for the low proportion of complete cTNM staging of BC patients before initial treatment: 1) Incomplete staging examinations before the initial treatment; 2) Lack of MDT discussions before the initial treatment; 3) Lack of thorough evaluation of the patients due to intensive workload for physicians; 4) Incompetent medical record-keeping despite complete evaluation by the physicians; 5) Lack of effective monitoring mechanism on the information system of the hospital. The low standardization rate of chemotherapy records for BC patients was due to the following factors: 1) Inattention to standardized chemotherapy records by the physician; 2) Inadequate training for the physician on the application of standardized chemotherapy records; 3) Inadequate training on standardized documentation of chemotherapy records by the hospital; 4) Dysfunctional medical information systems which ineffectively support the standardized management of chemotherapy records. The causes of the low rate of rational use of anti-tumor drugs in BC patients were identified as follows: 1) Insufficient prescription review feedback from pharmacists; 2) Poor communication and collaboration between pharmacists and clinicians, resulting in missed opportunities for prompt identification and resolution of drug misuses; 3) Lack of effective supervision and management measures by the hospital on the use of anti-tumor drugs; 4) Inadequate knowledge by the physicians on the necessary standards for rational use of anti-tumor drugs.

#### 3.3.2 Implementation of countermeasures

**Figure 3:**
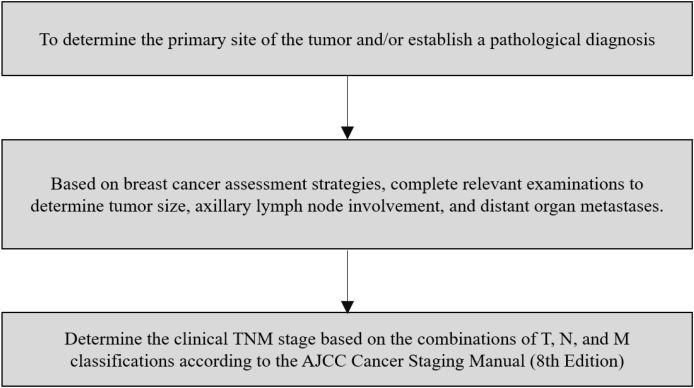
Clinical TNM staging workflow for breast cancer.

To address the causes of insufficient deficiencies in the quality control indicators for neoadjuvant and adjuvant therapy for BC, the Breast Cancer Neoadjuvant and Adjuvant Therapy Management Group developed the following countermeasures:

1. Enhancing the Proportion of Complete cTNM Staging of BC Patients Before Initial Treatment:

•& Strengthen the Management of the cTNM Staging Evaluation Process: The management group established and improved the tumor cTNM staging evaluation process by ensuring that physicians comprehensively consider patients’ medical history, imaging data, and other relevant information to accurately assess tumor development and progression. A detailed assessment process is outlined in **Figure 3**.

• Master Assessment Strategies: The physicians were trained and supervised to master the assessment strategies for cTNM staging for BC patients before treatment. Strategy One: (Breast ultrasound or breast X-ray (mammography) or breast MRI) + Chest CT + Abdominal ultrasound (or CT or MRI). Strategy Two: (Breast ultrasound or breast X-ray (mammography) or breast MRI) + PET/CT.

• Improve Medical Record Documentation: Physicians were trained and supervised to facilitate the standardization of the documentation of cTNM. A comprehensive pre-treatment diagnosis included a cTNM staging assessment. Additionally, if staging could not be determined post-examination, “x” was used to indicate uncertainty, with a maximum of one “x” (e.g., breast malignancy, cT_2_N_x_M_1_). The cTNM staging was documented in admission records, medical records (including the first medical record), or pre-treatment informed consent forms (e.g., surgical, anti-tumor drug treatment, and radiotherapy consent forms).

• Promote Tumor MDT Treatment Model: The MDT diagnosis and treatment model was actively implemented, a breast cancer MDT expert panel was established, and a multidisciplinary collaborative diagnosis and treatment was adopted for BC patients (especially for newly diagnosed patients) to ensure the development of scientific and standardized clinical TNM staging evaluation and treatment plan.

• Strengthen Clinical TNM Staging Evaluation Training: The management group regularly organized targeted training and retraining for medical staff in clinical departments, focusing on the principles, methods, and guidelines of TNM staging in BC. This approach improved the accuracy of clinical TNM staging, thereby enhancing the standardization and uniformity of BC care.

• Enhance Informatization Monitoring: The hospital information system was enhanced by incorporating additional data fields such as “status of the first anti-tumor treatment” and “TNM staging” into the electronic medical record.

2. Enhancing the Standardization Rate of Chemotherapy Records in BC Patients:

• Establish Standards for BC Chemotherapy Records: Develop standardized guidelines for chemotherapy records based on QCISDTBC-2022.^5^ Chemotherapy records included the following: chemotherapy regimens, chemotherapy drug doses, chemotherapy duration, and detailed records of important information such as patient height, weight, body surface area (BSA), standard application dose, actual application dose, dose calculation basis, route of administration, and pretreatment (**Table 2**). All chemotherapy patients strictly followed this standard for records.

**Table 2:** Example of a standardized chemotherapy record.

Diagnosis	Right-sided breast cancer, pTNM: T_2_N_0_M_0_
Pathology	Invasive Breast Cancer
Immunohistochemistry	HER2 (-), ER (-), PR (-)
Treatment Plan	AC–T (Epirubicin + Cyclophosphamide, q21d, 4 cycles, followed by sequential Docetaxel, q21d, 4 cycles)
Current treatment drugs and medication time	Epirubicin 150 mg IV infusion on day 1 + Cyclophosphamide 1.0 g IV infusion on day 1
Dose calculation	The patient is female, with a height of 160 cm, weight of 61 kg, and BSA of 1.66 m². The theoretical dose of Epirubicin is 90–100 mg/m² × 1.66 m² = 149.4–166 mg, with an actual dose of 150 mg. The theoretical dose of Cyclophosphamide is 600 mg/m² × 1.66 m² = 996 mg, with an actual dose of 1000 mg.
Treatment Interval	One cycle every 21 days
Combination Therapy	Anti-emetic and prophylactic leukocyte stimulation therapy
Observational Indicators	Tumor markers, complete blood count (CBC), liver and kidney function tests, echocardiogram (ECHO), electrocardiogram (ECG), and full-body CT scan, etc.

TNM, tumor-node-metastasis; HER2, human epidermal growth factor receptor 2; ER, estrogen receptor; PR, progesterone receptor; IV, intravenous; BSA, body surface area.

• Utilize Information Systems to Standardize Medical Record Documentation: We developed and implemented an electronic template for BC chemotherapy records, alongside a standardized template for same-day chemotherapy documentation. These tools aimed to streamline clinical operations and ensure consistency in clinical documentation.

• Organize Training on Chemotherapy Record Documentation: Regular training sessions were conducted on the required standards for chemotherapy record documentation to enhance the professional skills and improve the medical record-writing practices of the physicians.

• Establish a Supervisory Mechanism: The management group conducted monthly on-site medical record supervision and quality control to confirm consistent updates of the chemotherapy records per the established regulations. Additionally, quality control issues were promptly communicated to relevant physicians for accurate updates.

3. Enhancing the Rate of Rational Use of Anti-Tumor Drugs in BC Patients:

• Develop Clinical Application Guidelines and Standards: The expert panel formulated institutional guidelines and application standards for the clinical use of anti-tumor drugs in neoadjuvant and adjuvant therapy for BC.

• Conduct Specialized Reviews of Treatment Plans: Targeted reviews of neoadjuvant and adjuvant anti-tumor treatment plans for breast cancer patients were conducted. Notably, the review results were audited by the BC expert panel and publicly disclosed throughout the hospital. A notification, appeal, and reward/penalty mechanism was established.

• Utilize Pre-Authorization Software for Information-Based Interception: A pre-prescription review software was implemented to intercept irrational prescriptions and medical orders.

• Organize Training and Assessment: Clinical and pharmaceutical experts in BC were consulted to conduct training and assessment on the rational use of anti-tumor drugs for medical staff engaged in BC diagnosis and treatment.

• Standardizing Off-Label Use of Anti-Tumor Drugs: A directory for off-label use of anti-tumor drugs in BC was established. This directory was developed based on evidence from drug labels in other countries or regions, international authoritative guidelines, clinical practice standards issued by national associations, and clinical pathways. Specialized reviews of off-label drug use for BC were conducted to standardize the application of these medications in the treatment of patients.

Notably, these measures were implemented and have been continuously improved since December 2022.

#### 3.3.3 Effectiveness confirmation and standardization of measures

Statistical analyses of the BC patients (n=208) at Jiangsu Cancer Hospital who underwent either a neoadjuvant or adjuvant therapy from September to November 2023 revealed the following on the quality control indicators of BC: The proportion of complete cTNM staging of BC patients before initial treatment increased from 92.50% before quality control management to 98.08% after its implementation. The standardization rate of chemotherapy records in BC patients improved by 66.65% after quality control management. Additionally, the rate of rational use of anti-tumor drugs in BC patients increased from 78% before quality control to 87.5% afterward, showing significant improvement (**Table 1**). These results demonstrate that the BC quality control indicator management model can enhance indicators for neoadjuvant and adjuvant treatment, further improving the standardization of neoadjuvant and adjuvant BC therapies and promoting the rational use of anti-tumor medications.

**Figure 4:**
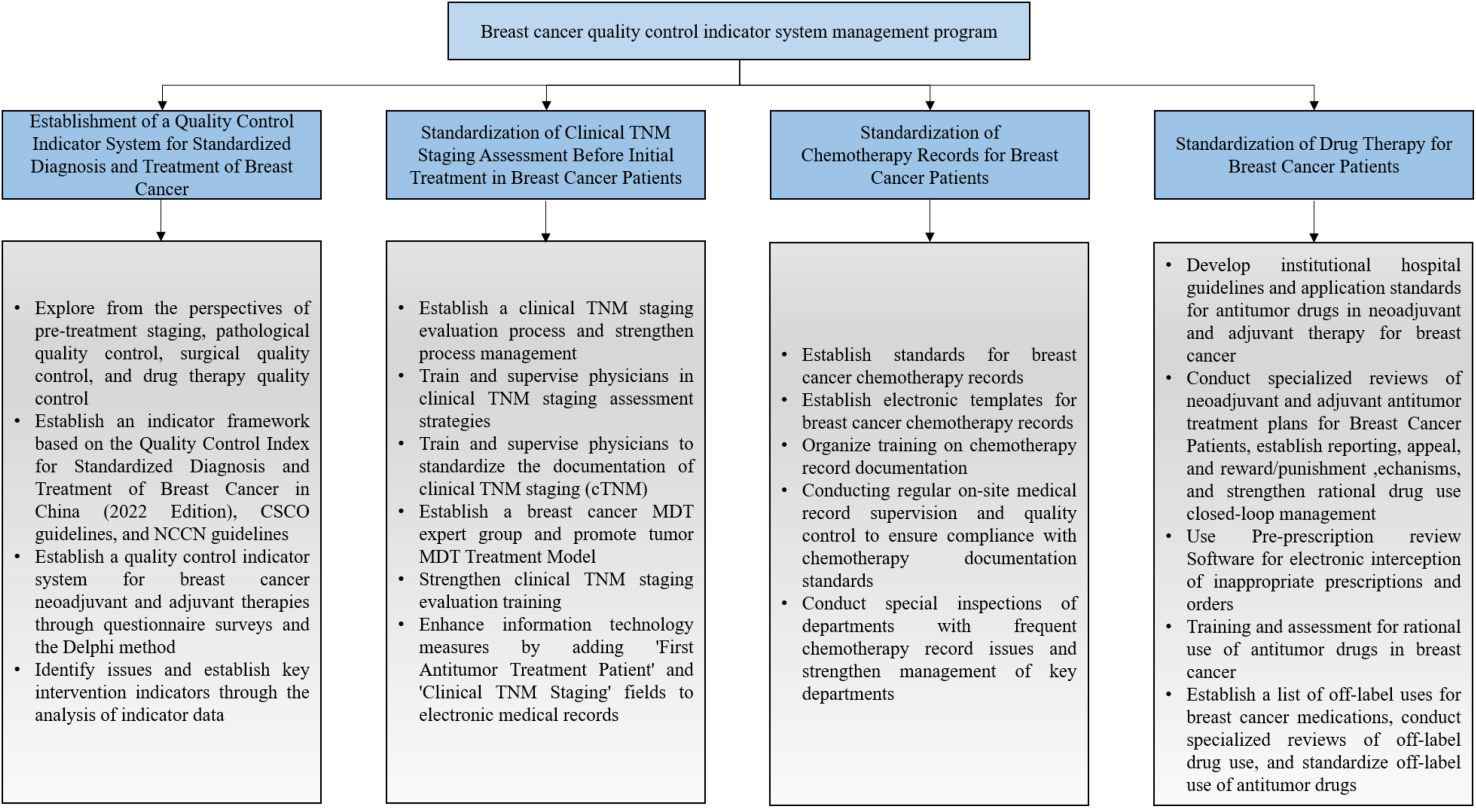
Standardization of measures in the quality control indicator system management program for breast cancer.

The strategies within the QCIS program were standardized and formulated into a protocol (**Figure 4**) to ensure consistency in future implementation and to facilitate their broader dissemination.

#### 3.3.4 Comparison of post-management data between the two cancer hospitals

Statistical analysis of BC quality control data from Jiangsu Cancer Hospital (Group A and Group A’ in **Figure 2**) and Nantong Tumor Hospital (Group B and Group B’ in **Figure 2**) for patients who received either neoadjuvant or adjuvant therapy between July to September 2022 and September to November 2023 was conducted. Additionally, the improvements in various QC indicators before and after management between these two centers were compared. The results indicated that the proportion of complete cTNM staging before initial treatment in Group A’ was increased by 5.58% compared to Group A; conversely, it decreased by 0.5% in Group B’, compared to Group B. The standardization rate of chemotherapy records in Group A’ increased by 66.65% compared to Group A, whereas in Group B’, it decreased by 16% compared to Group B. The rational use rate of anti-tumor drugs in Group A’ increased by 9.5% compared to Group A, while in Group B’, it decreased by 9.5% compared to Group B (**Table 1**). When comparing the magnitude of change between hospitals, Jiangsu Cancer Hospital demonstrated significantly greater improvements than Nantong Tumor Hospital in the standardization rate of chemotherapy records (Δ +66.65% vs. −16.00%, P < 0.001), the rate of rational use of anti-tumor drugs (Δ +9.5% vs. −9.5%, P < 0.001), and the completeness of cTNM staging before treatment (Δ +5.58% vs. 0.00%, P = 0.027) (**Table 3**). No significant between-hospital differences in Δ rates were observed for the other indicators. These results demonstrated that the BC quality control indicator management model can improve management quality beyond the capabilities of the existing conventional management system, further enhancing the standardization of BC diagnosis and treatment.

**Table 3:** Exploratory clinical outcomes and patient-reported outcomes before and after implementation of the Quality Control Indicator System program in Jiangsu Cancer Hospital and during the corresponding observation period in Nantong Tumor Hospital.

Outcome category	Indicator	Jiangsu cancer hospital		Nantong tumor hospital	
		Baseline data (group A: n=200)	Post-management data (group A’: n=208)	Baseline data (group B: n=200)	Post-management data (group B’: n=200)
Clinical outcome	Proportion of patients achieving pathological complete response (pCR) ^a^	32.76% (19/58)	40.91% (9/22)	37.50% (6/16)	33.33% (6/18)
	Proportion of patients remaining disease-free at 2 years (2-year DFS) ^b^	96.43% (189/196)	97.54% (198/203)	96.88% (186/192)	95.79% (182/190)
Patient-reported symptoms ^c^	Fatigue (%)	24.50% (49/200)	17.31% (36/208)	22.00% (44/200)	19.00% (38/200)
	Nausea/vomiting (%)	51.00% (102/200)	42.79% (89/208)	52.50% (105/200)	54.50% (109/200)
	Diarrhea (%)	8.00% (16/200)	7.21% (15/208)	4.50% (9/200)	6.00% (12/200)
	Precordial discomfort/chest tightness (%)	1.50% (3/200)	0.48% (1/208)	0.50% (1/200)	2.50% (5/200)
	Peripheral neuropathy (%)	12.00% (24/200)	9.13% (19/208)	14.00% (28/200)	16.00% (32/200)
Other treatment-related toxicities	Myelosuppression (%) ^d^	39.50% (79/200)	31.73% (66/208)	33.50% (67/200)	25.50% (51/200)
	Liver damage (%) ^e^	3.50% (7/200)	4.81% (10/208)	6.00% (12/200)	7.00% (14/200)
Treatment adherence	Proportion of patients completing planned neoadjuvant therapy (%) ^f^	80.25% (65/81)	89.74% (35/39)	83.87% (26/31)	84.44% (38/45)
	Proportion of patients completing planned adjuvant therapy (%) ^g^	84.87% (101/119)	91.12% (154/169)	89.94% (152/169)	88.39% (137/155)

a The denominator was the number of patients who received neoadjuvant therapy and had documented chemotherapy response assessment after surgery during the same period.

b 2-year DFS was defined as the proportion of patients without recurrence or death within 24 months from the date of definitive surgery. The denominator included patients with available 2-year follow-up data, excluding those lost to follow-up before 24 months.

c Patient-reported symptoms were retrospectively extracted from medical records and follow-up documentation and defined as the presence of the corresponding symptom of any grade during treatment.

d Myelosuppression was defined as the occurrence of grade ≥2 neutropenia, anemia, or thrombocytopenia according to CTCAE version 5.0.

e Liver damage was defined as the presence of grade ≥2 elevations in ALT, AST, or bilirubin according to CTCAE version 5.0.

f Neoadjuvant therapy completion referred only to planned neoadjuvant chemotherapy and/or targeted therapy; neoadjuvant endocrine therapy was not included.

g Adjuvant therapy completion referred only to adjuvant chemotherapy and/or targeted therapy. Endocrine therapy was excluded because its prolonged duration precluded complete follow-up within the study period.

### 3.4 Clinical outcomes, patient-reported outcomes, and treatment adherence

Exploratory clinical outcomes, patient-reported symptoms, and treatment adherence before and after QCIS implementation at Jiangsu Cancer Hospital, as well as during the corresponding observation period at Nantong Tumor Hospital, are summarized in **Table 3**. In Jiangsu Cancer Hospital, the pCR increased from 32.76% at baseline to 40.91% after implementation, whereas in Nantong Tumor Hospital, pCR showed a slight decrease from 37.50% to 33.33%. The 2-year DFS rate remained high and stable in both hospitals, with values exceeding 95% across all groups.

With respect to patient-reported symptoms, modest reductions were observed in Jiangsu Cancer Hospital after QCIS implementation, including fatigue (from 24.50% to 17.31%), nausea/vomiting (from 51.00% to 42.79%), diarrhea (from 8.00% to 7.21%), precordial discomfort/chest tightness (from 1.50% to 0.48%), and peripheral neuropathy (from 12.00% to 9.13%). In Nantong Tumor Hospital, symptom rates showed relatively small fluctuations over the corresponding observation period, without a consistent pattern of improvement. Among other treatment-related toxicities, myelosuppression decreased in both hospitals (Jiangsu: from 39.50% to 31.73%; Nantong: from 33.50% to 25.50%), whereas liver damage remained infrequent and relatively stable in both groups.

Regarding treatment adherence, the proportion of patients completing planned neoadjuvant therapy increased in Jiangsu Cancer Hospital from 80.25% to 89.74%, and completion of planned adjuvant therapy improved from 84.87% to 91.12%. In Nantong Tumor Hospital, completion rates for neoadjuvant and adjuvant therapy remained comparable (83.87% vs. 84.44% and 89.94% vs. 88.39%, respectively).

Overall, although several clinical, symptom-related, and adherence indicators demonstrated favorable trends in the intervention hospital following QCIS implementation, none of the within-hospital pre–post comparisons reached statistical significance based on chi-square testing (all P > 0.05). These findings suggest potential improvements in clinical practice patterns and patient experience associated with the management program, while highlighting that the exploratory nature and limited sample sizes of these outcomes preclude definitive conclusions regarding statistical effectiveness.

### 3.5 Qualitative insights from clinicians and patients

From the clinician perspective, several facilitators were noted, including clearer definitions of chemotherapy protocols, improved coordination among medical oncology, surgery, pathology, and pharmacy, and enhanced standardization of documentation following QCIS implementation. Clinicians reported that structured templates and strengthened audit mechanisms reduced ambiguity in treatment decision-making and supported more consistent adherence to guidelines. Challenges included increased documentation workload during early implementation and the need for repeated training to ensure compliance, particularly among newly onboarded staff. Variability in baseline practice across departments also contributed to uneven adoption of standardized processes.

From the patient perspective, follow-up notes reflected concerns about treatment-related fatigue, nausea/vomiting, myelosuppression, anxiety about recurrence, and uncertainty about treatment outcomes. Nevertheless, many patients described positive experiences, including clearer explanations of treatment plans, improved communication with clinical staff, and smoother transitions between diagnostic evaluation, surgery, and systemic therapy. These changes contributed to perceived improvements in coordination and confidence in care quality.

These qualitative observations offer additional insights into the facilitators and barriers encountered during implementation and help contextualize the observed improvements in quality control indicators.

## 4 Discussion

Based on recent data, the 5-year relative survival rate for cancer in China has improved from 30.9% to 40.5% (10). However, this figure remains considerably lower than the 67.9% reported in the United States (11). This disparity may be attributed to prolonged delays in patients seeking medical consultation, regional disparities in diagnostic and therapeutic capabilities, and suboptimal adherence to established cancer diagnostic and treatment guidelines (12–14). Consequently, there is an urgent need to enhance the quality, standardization, and uniformity of cancer diagnosis and treatment practices in China. To address these issues, NCQCC was established in 2012 and is responsible for the quality control and improvement of the standardized diagnosis and treatment of tumors nationwide. Since 2020, comprehensive quality control for single cancer types has been fully established, with BC serving as the first pilot tumor. BC quality control protocols are expected to play a leading role in enhancing the overall quality of cancer prevention and treatment in China.

Following the implementation of the BC quality control indicator system management program, key intervention indicators at Jiangsu Cancer Hospital—including the proportion of complete cTNM staging prior to initial treatment, the standardization rate of chemotherapy records, and the rate of rational use of anti-tumor drugs—showed significant improvements, whereas the majority of indicators at Nantong Tumor Hospital showed no improvement or a decline during the same period. When comparing the magnitude of change between hospitals, Jiangsu Cancer Hospital demonstrated significantly greater improvements in these key indicators, while no significant between-hospital differences were observed for the remaining measures (**Table 3**). It should be noted that the two hospitals differed substantially in baseline performance, and hospitals with higher baseline standardization rates may experience smaller observable improvements due to ceiling effects, whereas institutions with lower baseline performance may demonstrate larger relative gains following structured quality improvement interventions. Therefore, inter-hospital comparisons should be interpreted with caution, with greater emphasis placed on within-hospital changes over time. The decline observed at Nantong Tumor Hospital may reflect the inherent sensitivity of documentation-dependent, process-based indicators to variations in case complexity, workload distribution, and record completeness, particularly in the absence of a structured quality control framework with continuous audit, feedback, and reinforcement mechanisms. Such fluctuations have been reported in quality assessment studies when standardized monitoring systems are not systematically implemented, underscoring the importance of sustained quality governance rather than isolated performance measurements.

Rational use of antineoplastic drugs is fundamental to the standardized management of breast cancer, directly influencing both therapeutic efficacy and patient safety (15–17). Traditional assessments of rationality rely primarily on concordance with authoritative guidelines (e.g., NCCN, CSCO) and drug package inserts; however, defining “rational use” in real-world clinical practice is increasingly complex. The rapid evolution of oncology evidence, delays in updates to clinical guidelines and regulatory labels, and patient-specific factors—such as comorbidities, age, frailty, and urgent therapeutic needs—may create situations in which off-guideline or off-label treatment choices reflect clinically justified decisions rather than inappropriate practice.

Off-label use of anticancer drugs has been widely documented across oncology (18, 19). A systematic review reported that 13%–71% of adult cancer patients receive at least one off-label chemotherapy prescription (20), most commonly due to use in unapproved tumor indications or modified applications. Importantly, the proportion of off-label use unsupported by clinical guidelines or drug compendia ranged from 7% to 31%, indicating that a substantial fraction of off-label prescribing is evidence-informed despite lacking formal regulatory approval. In BC specifically, large retrospective cohort analyses from the United States have shown that more than half of anticancer therapy encounters were off-label at the time of use, although the majority were supported by emerging clinical evidence or guideline recommendations (21). Similarly, a study in elderly breast cancer patients demonstrated that while 75% of prescriptions were classified as “inappropriate” under strict regulatory definitions, 64% were nevertheless concordant with NCCN guidelines (22), underscoring the ambiguity between formal approval status and contemporary clinical evidence. Collectively, these observations suggest that off-label use in oncology is not inherently irrational, but rather reflects the dynamic interplay between evolving evidence, regulatory processes, and individualized clinical decision-making.

In this study, rationality was assessed using dimensions directly relevant to the safety and efficacy of systemic antineoplastic therapy, including regimen selection, dosage accuracy, solvent preparation, drug sequencing, and adherence to recommended treatment duration. While guideline adherence served as the benchmark, the clinical context of deviations was reviewed to distinguish justifiable individualization from clearly inappropriate practices. Several instances of inappropriate use were identified, such as administration of adjuvant chemotherapy to low-risk hormone receptor (HR)+/HER2− patients without indications (e.g., Ki-67 <5%, tumor <2 cm, N0). Deviations in regimen selection were also observed. Examples included the use of TCHP (docetaxel + cyclophosphamide + trastuzumab + pertuzumab) or TAC (docetaxel + epirubicin + cyclophosphamide) for HER2-positive neoadjuvant treatment despite recommendations favoring TCbHP (paclitaxel + carboplatin + trastuzumab + pertuzumab), THP (paclitaxel + trastuzumab + pertuzumab), and TH (docetaxel + trastuzumab) combined with pyrotinib, or AC-THP (anthracycline + cyclophosphamide, followed by paclitaxel + trastuzumab + pertuzumab); administration of NX (vinorelbine + capecitabine) as adjuvant therapy for low-risk triple-negative breast cancer (TNBC) instead of standard anthracycline–taxane regimens; use of XHP (capecitabine + trastuzumab + pertuzumab) as adjuvant therapy in HER2-positive disease, which is not guideline-supported; concurrent endocrine therapy with dual-targeted agents (e.g., trastuzumab + pertuzumab + exemestane; trastuzumab + pertuzumab + goserelin) in HR+/HER2+ adjuvant settings, outside of clinical trials, where such combinations are not supported by current guidelines or high-level evidence; and assignment of docetaxel–carboplatin as adjuvant therapy for high-risk TNBC, even though anthracycline–taxane regimens remain standard of care. Additional issues included under-dosing of key agents such as cisplatin, epirubicin, and cyclophosphamide, non-compliant dosing intervals (e.g., splitting epirubicin doses), and improper solvent or dilution practices.

The off-label use of PLD in our cohort exemplifies the nuanced distinction between “off-label” and “irrational” use. While NCCN guidelines primarily recommend PLD for metastatic breast cancer (7), its incorporation into neoadjuvant or adjuvant regimens may be clinically motivated, particularly for patients at heightened risk of anthracycline-induced cardiotoxicity. PLD maintains antitumor efficacy comparable to epirubicin while significantly reducing cardiac toxicity (23, 24). Evidence from the GeparOcto trial demonstrated that PLD-containing regimens achieved pCR and survival outcomes comparable to intensive anthracycline–taxane regimens in high-risk and TNBC populations (25, 26). Similarly, a study of 274 patients receiving neoadjuvant chemotherapy reported no significant difference in pCR rates between the PLD and epirubicin groups (11.6% vs. 7.0%, p=0.4578) (27). Likewise, the Opti-HER HEART study reported a 56.6% pCR rate with low cardiotoxicity in HER2-positive disease treated with PLD-based regimens (28). Studies have shown that adjuvant chemotherapy for breast cancer significantly reduces the risk of recurrence and prolongs the survival of patients (29, 30). Retrospective analyses of adjuvant therapy similarly suggest no significant differences in DFS or OS between PLD and epirubicin (31, 32), though sample sizes remain limited. Collectively, available data tend to indicate that PLD substantially lowers the risk of cardiotoxicity compared with conventional anthracyclines (23, 33, 34). Thus, for elderly patients or those with a history of chest irradiation, preexisting cardiovascular disease, cachexia, or pericardial involvement, PLD may represent a clinically appropriate alternative in the neoadjuvant or adjuvant setting to reduce cardiotoxicity risk. For patients without such risk factors, conventional anthracyclines should remain the preferred option in accordance with guideline-recommended practice.

Taken together, these findings highlight two important considerations. First, strict guideline adherence alone may not fully capture the appropriateness of clinical decisions, especially in rapidly evolving therapeutic landscapes. Incorporating contextual elements—such as patient comorbidities, prior toxicity, and multidisciplinary expert input—is essential for accurately interpreting deviations from guidelines. Second, quality improvement programs that monitor rational drug use should balance regulatory requirements with clinical flexibility, promoting both safety and evidence-based innovation. Future work should include collecting structured information on the clinical rationale for off-label or non-guideline use and integrating clinician-reported decision pathways. Additionally, multicenter studies with prospective data collection will help better define the boundaries between necessary individualized care and unwarranted practice variation.

To the best of our knowledge, this study is the first to apply a QCIS management model to improve the diagnosis and treatment of single tumor types. The results indicate that adoption of the QCIS management program will promote the standardization of breast cancer diagnosis and treatment and enhance the rational use of medications. While the findings of this study are promising, several limitations must be acknowledged. First, this study was conducted in only two hospitals within Jiangsu province, with the QCIS intervention implemented at Jiangsu Cancer Hospital and Nantong Tumor Hospital serving as a contemporaneous comparison center. The two institutions differed in baseline performance, organizational structure, and resource availability. These contextual differences may limit the generalizability of the observed intervention effects to other hospitals or healthcare systems; therefore, the results should be interpreted with appropriate caution. In addition, the substantial heterogeneity between the two institutions may influence the magnitude of the observed effect, underscoring the need for further validation in broader and more diverse settings. Second, the study utilized a retrospective and non-randomized design. Although a contemporaneous comparison hospital was included, the assignment of hospitals to intervention versus comparison groups was not randomized, potentially introducing selection bias at both the institutional and patient levels. While such designs are common in real-world quality improvement initiatives, they cannot fully address unmeasured confounding. Third, the relatively short follow-up duration hindered the assessment of longer-term clinical outcomes, such as overall survival or 5-year DFS. Fourth, although several exploratory clinical and patient-reported outcomes were included, qualitative insights from clinicians and patients were not systematically collected. Instead, retrospective qualitative data from routine clinical records were used to contextualize the quantitative findings and identify barriers and facilitators to the QCIS program’s effectiveness. Future research should focus on prospective or multicenter studies with more diverse hospital settings, longer follow-up periods, and mixed-methods approaches to provide more robust evidence and enhance the external validity of the QCIS.

## 5 Conclusion

This is the first study to construct and implement a quality control indicator system management program aimed at improving the standardization of neoadjuvant and adjuvant therapies in the treatment of breast cancer. The proposed program effectively enhanced key quality indicators at the Jiangsu Cancer Hospital, including the proportion of complete clinical TNM staging before initial treatment, the standardization rate of chemotherapy records, and the rate of rational use of anti-tumor drugs. In contrast, these benefits were not observed for the control hospital, demonstrating the program’s superiority over routine management practices. These findings demonstrate that the structured quality control system can improve the quality and consistency of breast cancer diagnosis and treatment. Future large-scale, multicenter studies are needed to validate our results and explore the broader clinical benefits of the proposed program.

## Data Availability

The raw data supporting the conclusions of this article will be made available by the authors, without undue reservation.
